# Discrete hypo/de-pigmented spots as an early, prodromal, objective sign of somatic origin pain

**DOI:** 10.1097/PR9.0000000000000581

**Published:** 2016-12-15

**Authors:** Kritika Doshi

**Affiliations:** Pain Clinic in Charge, Jupiter Hospital, Thane, India. E-mail address: kritikadoshi@hotmail.com.

***Letter To Editor:***

I am a clinician working in a private set-up in India. I would like to draw attention to a common observation seen on the skin of patients presenting to the pain clinic. Many of the patients have discrete, 1–4 mm in diameter, hypo/de-pigmented spots on the skin surface. Although these are obviously visible, the patients themselves were not aware of their existence or the time of origin of the spots. This is not an isolated finding but a recurring observation seen in patients presenting to the pain clinic. Because this is a new finding, I will refer to them as KD-spots.

The presence of KD-spots is visible because of the contrast between the hypopigmented spot and pigmented skin of our population when the patient is lying on the examination table. These spots were seen commonly in senior citizens in multiple dermatomes (almost everyone has some). In the pain clinic, the youngest patient who had these spots was 19 years old.

To verify that the spots were not an isolated or random occurrence and could be seen in diverse pain conditions, 710 patients (with visible spots) of all ages presenting to the pain clinic were included. Inclusion criteria included patients with complaints of various chronic pains: (1) 399 with back, neck, and upper and lower limb pain (ie, nonradicular pain, “tennis elbow,” and shoulder/knee pain); (2) 185 with persistent pain after surgery (hernia repair, knee replacement, lower section caesarean section, open cholecystectomy, and spine); (3) 48 with nonvisceral abdominal pain; and (4) 78 with cancer and pain. The numeric rating scale (NRS) score varied from 2 to 7. The pain was predominantly “aching” (they described the pain in Hindi as “mere haath pav toot rahe hai” translated as my hands and legs are breaking); none of the patients had severe pain but their activities of daily living were affected.

I have observed that the presence of KD-spots does not seem to be a random occurrence. They seem to be present in a dermatomal distribution. To look for evidence of a probable prediction and identification of somatic origin of the pain, the distribution of patients' hypo/de-pigmented visible spots was corelated to the corresponding intervertebral disk magnetic resonance imaging (MRI) images of the spine (to look for disk prolapse/annular tear/degeneration). Sixteen patients with back pain did not have the MRI at first visit and were lost to follow-up; hence, corelation could not be performed in these patients. (1) Three hundred thirty-eight of 399 patients (84.71%) presenting with back/neck and limb pain had visible spots present in the same dermatome (corresponding to the degenerated disk on MRI). The MRI corelation could be performed in 383 of these patients. (2) One hundred twelve of 185 patients (80.51%) with persistent postsurgical pain had KD-spot in the following distributions: T8/9 dermatome in postcholecystectomy, T12-L1 distribution in posthernia surgery, and patients with lower section caesarean section. L2,3,4 dermatomes in postknee replacement, spine surgery had spots ventrally and dorsally over L2-3-4-5, 30 of 48 patients presenting with “abdominal pain” had visible KD-spots on anterior abdominal wall, 60 of 78 patients with cancer had KD-spots in various dermatomes unrelated to the primary disease. However, the MRI showed degenerative changes in the intervertebral disk corresponding to dermatome of the KD-spots.

A dermatologist consultation to rule out other possible causes of hypo/depigmentation was undertaken for initial 4 patients who ruled out infective etiology and gave a diagnosis of “idiopathic hypomelatonic dermatosis” (Asymptomatic, macular, depigmented lesions seen commonly after the age of 40).

There is no evidence or report of neither similar observations nor explanation to the presence or etiology of these spots. More research is needed to verify the etiology and significance if any of these spots. There are reports describing the morphology and receptive fields of C-polymodal afferent fibers, which are reminiscent of the spots visible on the skin; Christenson and Perl in 1970 and Schouenborg and Sjolund in 1983 have reported that many neurons, driven by C fibers in laminae I and II have small receptive fields on the skin. Various studies have defined the receptive field properties of unmyelinated tactile afferents in the human skin^4,8^ and the morphological features of nociceptive C-afferents from muscle.^1–3,5^ Chemical sensitization of dorsal root ganglion neurons occurs with release of nociceptive substances by the disk.^6^ It is speculated that leakage of nociceptors from the disk sensitizes the C or A delta unmyelinated nerve fibers in the annulus and the dorsal root ganglion, lowering the nociceptor threshold for mechanical stimulation.^7^ The findings in the abovementioned references very much correlate with the observed spots on the corresponding skin of the affected intervertebral disk (Somatome involved), at least for the patients complaining of radicular pain.

I would like to propose a hypothesis that these spots indicate an objective sign of early internal disc disruption, leak of nuclear material causing sensitization of the corresponding somatic nerve with distal effects on 1 or all components of the somatome presenting clinically as KD-spots in the dermatome (representing the visible evidence of the receptive field of polymodal C-afferent fibres), myofascial taut bands/trigger points in the myotome and ‘bone’ or ‘joint’ pain in scelrotome. Hypo/depigmentation is caused by apoptosis/cellular destruction of the cell body secondary to immunologic/inflammatory events after leakage of nucleus pulposus and sensitization of dorsal root ganglion neurons (based on evidence of melatonin levels and disk degeneration; autophagy) (Figure 1, 1–4mm diameter, discrete, hypopigmented spots).

**Figure 1.**
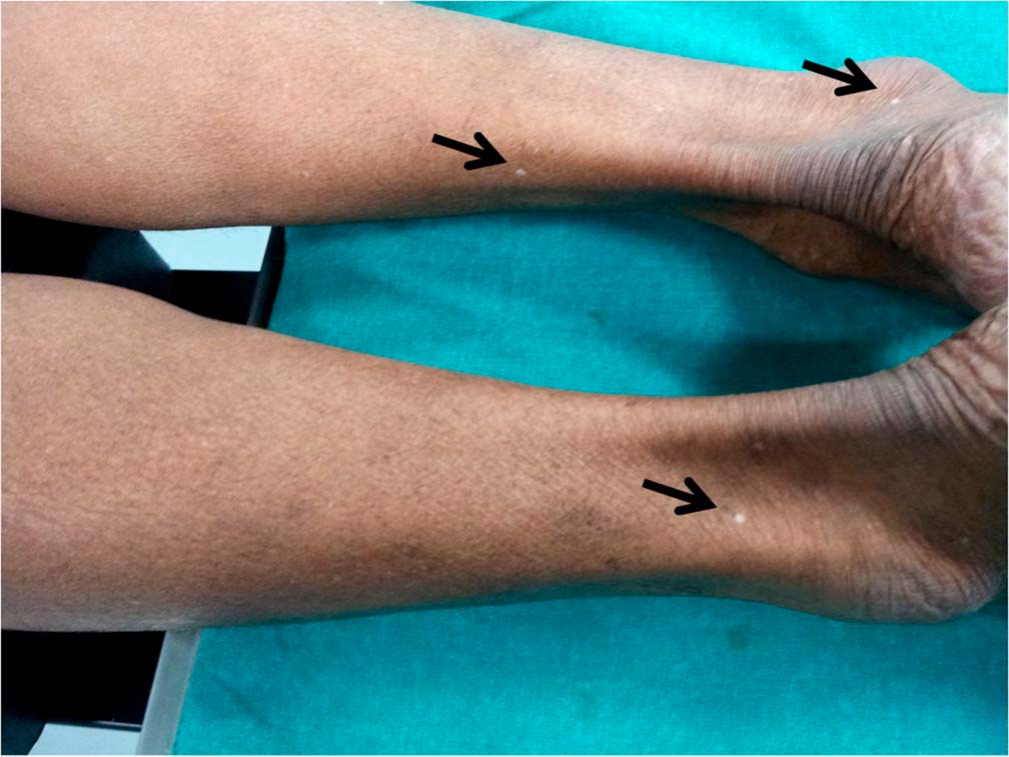
Visible discrete, hypopigmented KD Spots over L5, S1 dermatomes.

## Conflict of interest statement

The author has no conflicts of interest to declare.
